# Erratum to: The advantages of SMRT sequencing

**DOI:** 10.1186/s13059-017-1295-y

**Published:** 2017-08-16

**Authors:** Richard J. Roberts, Mauricio O. Carneiro, Michael C. Schatz

**Affiliations:** 1 New England Biolabs, 240 County Road, Ipswich, MA 01938 USA; 2 Broad Institute of Harvard and MIT, Cambridge, MA 02142 USA; 3 Simons Center for Quantitative Biology, Cold Spring Harbor Laboratory, Cold Spring Harbor, NY 11743 USA

## Erratum

It has been highlighted that the original manuscript [1] has been published in duplicate and indexed on the SpringerLink platform. The duplicate article [2] is not the official version of this manuscript. We apologise for any confusion caused by this discrepancy and acknowledge the former, original manuscript [1], also indexed on the PubMed platform, as the official version of this work on behalf of the author.

The publisher apologises for these errors.
